# Multiomics Research: Principles and Challenges in Integrated Analysis

**DOI:** 10.34133/bdr.0059

**Published:** 2024-12-05

**Authors:** Yunqing Luo, Chengjun Zhao, Fei Chen

**Affiliations:** ^1^ National Key Laboratory for Tropical Crop Breeding, College of Breeding and Multiplication, Sanya Institute of Breeding and Multiplication, Hainan University, Sanya 572025, China.; ^2^ College of Tropical Agriculture and Forestry, Hainan University, Danzhou 571700, China.

## Abstract

Multiomics research is a transformative approach in the biological sciences that integrates data from genomics, transcriptomics, proteomics, metabolomics, and other omics technologies to provide a comprehensive understanding of biological systems. This review elucidates the fundamental principles of multiomics, emphasizing the necessity of data integration to uncover the complex interactions and regulatory mechanisms underlying various biological processes. We explore the latest advances in computational methodologies, including deep learning, graph neural networks (GNNs), and generative adversarial networks (GANs), which facilitate the effective synthesis and interpretation of multiomics data. Additionally, this review addresses the critical challenges in this field, such as data heterogeneity, scalability, and the need for robust, interpretable models. We highlight the potential of large language models to enhance multiomics analysis through automated feature extraction, natural language generation, and knowledge integration. Despite the important promise of multiomics, the review acknowledges the substantial computational resources required and the complexity of model tuning, underscoring the need for ongoing innovation and collaboration in the field. This comprehensive analysis aims to guide researchers in navigating the principles and challenges of multiomics research to foster advances in integrative biological analysis.

## Multiomics Equipment

**Fig. 1.**
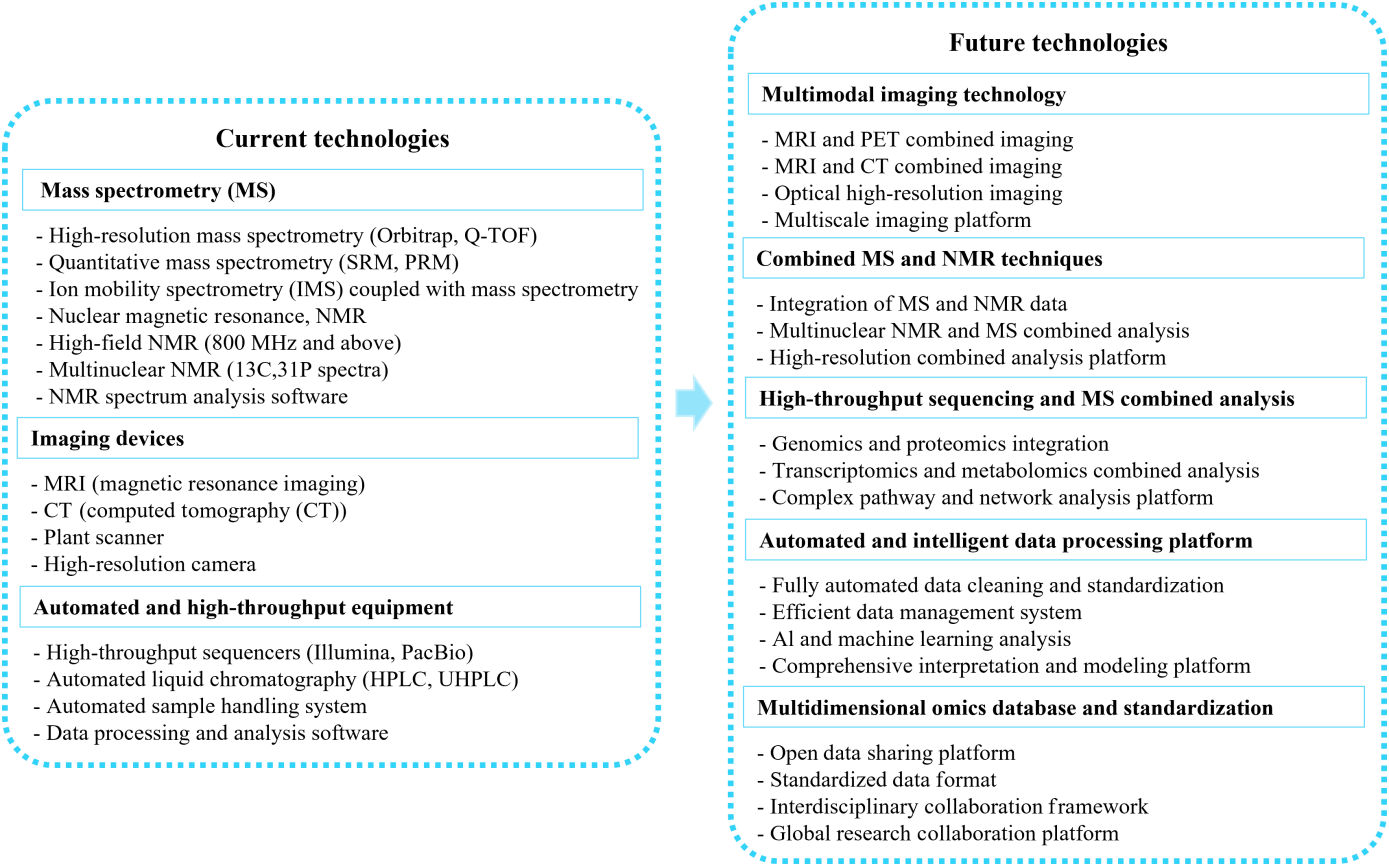
Current and prospective future multiomics technologies.

Multiomics research relies on advanced equipment to acquire, analyze, and interpret complex batches of biological data (Fig. 1). The advantages of this approach include higher data throughput, better batch consistency, and faster speed than traditional methods.

### High-throughput DNA sequencing equipment

Automated and high-throughput equipment plays a critical role in the acquisition of multiomics data, importantly advancing biological research. Illumina sequencers, Pacific Biosciences (PacBio) Revio sequencers, and Oxford Nanopore Technologies (ONT) sequencers are representative of the devices currently available. Each of these sequencers features unique innovative technologies and characteristics that make them stand out in various research fields.

Illumina sequencers are leaders in high-throughput sequencing [next-generation sequencing (NGS)] technology [1] and are widely used in genomics, transcriptomics, and epigenomics. They utilize reversible terminator chemistry and bridge amplification to generate high-density DNA clusters on flow cells, producing billions of reads per run. This approach importantly increases sequencing speed and data output. Owing to their high throughput and low error rates, Illumina sequencers are the preferred choice for mutation detection and precise genome assembly and are suitable for sequencing projects ranging from small genomes to large human genomes. The output data, typically in the form of short reads (150 to 400 base pairs) and formatted as FASTQ, are particularly well suited for sequence alignment and mutation detection.

PacBio Revio sequencers represent third-generation sequencing technology, which is based on single-molecule real-time (SMRT) sequencing [2]. They read sequences by observing DNA polymerase synthesis in real time. PacBio Revio sequencers are known for their ultralong read lengths and high accuracy, with single reads reaching tens of thousands of bases, averaging 10 to 15 kb. This importantly increases the quality of complex genome assemblies. With high-fidelity (HiFi) read technology, these sequencers combine high accuracy with long read lengths to excel in genome assembly and structural variation analysis. Additionally, PacBio Revio sequencers can directly detect epigenetic modifications such as DNA methylation [3]. Their typical data output formats are BAM and FASTQ.

ONT sequencers use nanopore technology to provide real-time, portable, and long-read sequencing solutions. By directly reading the sequences of DNA or RNA molecules as they pass through nanopores via changes in electric current, ONT sequencers achieve exceptional portability and real-time data output. Compact and portable devices, such as the MinION, can be used in field or clinical settings, allowing immediate data acquisition and rapid response during sequencing. ONT sequencers offer read lengths of up to hundreds of thousands of bases, making them suitable for whole-genome sequencing and transcriptome analysis [4]. Their typical data output formats are FASTQ and BAM.

High-throughput DNA library preparation instruments play a crucial role in modern genomic research by automating and optimizing the process of preparing DNA samples for sequencing. These instruments are designed to handle large numbers of samples simultaneously, ensuring efficiency, reproducibility, and scalability in genomic studies. Examples of high-throughput DNA library preparation instruments include the Illumina Nextera DNA Flex Library Prep Kit [5], which utilizes tagmentation technology for fast and efficient DNA library preparation; the Thermo Fisher Scientific Ion Torrent [6], which offers automated library preparation solutions for sequencing on Ion Torrent platforms; the Qiagen QIAseq FX Library Kit [7], which provides flexible library preparation protocols compatible with multiple sequencing platforms; and the ONT [8] automated library preparation workflows for their nanopore sequencing platforms, such as the MinION and PromethION.

### Mass spectrometry

Continual innovation is improving the ability of mass spectrometers to analyze proteins, metabolites, and other biomolecules in complex samples. Advances such as high-resolution mass spectrometry (HR-MS) (e.g., Orbitrap technology) and quadrupole time-of-flight (Q-TOF) MS have importantly increased resolution and sensitivity [9], enabling the detection of molecules at lower concentrations and the differentiation of isotope subtypes. The data produced by mass spectrometers typically include mass spectra and MS data files, which contain information on the mass-to-charge ratio (m/z) and the relative abundance of each analyte. High-throughput MS technology can generate large volumes of metabolite and protein analysis data, supporting large-scale biomarker discovery and quantitative analysis.

In metabolomics, advanced MS techniques such as HR-MS/MS and ion mobility spectrometry (IMS) [10] enable the precise identification and quantification of thousands of metabolites, revealing intricate and dynamic changes in metabolic networks within organisms. Innovations such as MS imaging (MSI) and single-cell metabolomics are pushing boundaries, allowing spatially resolved metabolic profiling and the analysis of metabolite distributions at the single-cell level. Additionally, nuclear magnetic resonance (NMR) spectroscopy with enhanced sensitivity and resolution is used for comprehensive metabolite profiling [11]. These advances facilitate a deeper understanding of metabolic pathways, biomarker discovery, disease mechanisms, and the effects of therapeutic interventions, importantly contributing to personalized medicine and systems biology.

### Nuclear magnetic resonance

NMR technology is continually improving in terms of magnetic field strength, probe coil design, and spectrum analysis methods. High-field NMR systems (such as those operating above 800 MHz) [12] offer heightened signal strength and resolution, allowing the analysis of more complex biological samples. The data produced by NMR provide detailed information about molecular structures and dynamic processes, such as chemical shifts and coupling constants. NMR technology can obtain quantitative information and chemical structures of metabolites, supporting research in metabolomics and structural biology.

In metabolomics, NMR is used to identify and quantify metabolites within organisms, study metabolic pathways, and explore metabolic regulatory mechanisms [13]. In structural biology [14], NMR is employed to analyze the 3-dimensional (3D) structures of proteins and nucleic acids, revealing their functions and interaction mechanisms.

### Imaging devices

Medical imaging technologies [such as magnetic resonance imaging (MRI) and computed tomography (CT)] and plant imaging devices (such as high-resolution cameras and plant scanners) are continually improving in terms of imaging resolution, scanning speed, and data processing methods. The new generation of equipment facilitates the more precise analysis of structural details and tissue functions. Imaging devices generate detailed images of tissue structure and organism morphology, which are typically stored and analyzed as image files (such as DICOM files) or 3D reconstruction models [15]. These data support the study of the anatomy and developmental processes of animals and plants.

In phenomics, imaging devices are used to capture the morphological structure and tissue characteristics of individuals and to explore the associations between genotypes and phenotypes. In plant science, imaging technology helps in the study of plant root structure, leaf morphology, and flower development and in the analysis of plant adaptation to environmental changes and growth responses.

### Prospects for instrument development

Current multiomics research faces challenges in the integration of instruments, but with technological advances and increasing research needs, the future holds promise for the integration and joint analysis of multiomics data. Potential future directions and technological trends include, first, multimodal imaging technology. One future direction is the integration of various imaging technologies, such as MRI, positron emission tomography (PET), CT, and high-resolution optical imaging technologies (e.g., fluorescence imaging), to achieve multimodal imaging of animals and plants [16]. This integration can provide more comprehensive and 3D information on structure and function within organisms, from the cellular level to entire organs. A second potential direction is the combination of MS and NMR technology. MS and NMR each have advantages in analyzing proteins, metabolites, and other biomolecules. Future developments could integrate these technologies, combining multinuclear NMR and high-resolution MS analyses to explore the structure and function of complex molecular components within organisms in depth. Third, high-throughput sequencing can be combined with MS analysis. Combining high-throughput sequencing and MS technologies can enable the integrated analysis of genomics, transcriptomics, and metabolomics data [17]. This combined analysis can reveal the relationships between gene expression regulation and protein expression, further elucidating functional pathways and metabolic networks within organisms. Finally, automated and intelligent data processing platforms will be developed. Future multiomics research will require more powerful automated equipment and intelligent data processing platforms. These platforms can integrate various types of data and efficiently perform data cleaning, standardization, and analysis, thereby enabling the management and comprehensive interpretation of large-scale data.

## Multiomics Data

### Genomic data

#### Retrieval and parsing of genomic data

Genomics studies the structure, function, and evolution of genomes, with a primary focus on DNA sequences and gene variations. Genomic data are obtained through various advanced sequencing technologies, including NGS and third-generation sequencing. NGS technologies, such as Illumina HiSeq, Illumina NovaSeq, and Ion Torrent, can read short DNA fragments with high throughput. Illumina HiSeq and NovaSeq are known for their high accuracy and high throughput, generating data volumes ranging from tens of gigabytes to hundreds of gigabytes per sample, with data formats including FASTQ, BAM, and VCF. Ion Torrent utilizes semiconductor technology to directly detect changes in hydrogen ion concentration during DNA synthesis, offering data at lower costs and faster speeds.

Third-generation sequencing technologies, such as PacBio SMRT and Oxford Nanopore MinION, can read longer DNA fragments, providing greater sequence continuity and integrity. PacBio SMRT technology detects the incorporation of fluorescently labeled nucleotides during DNA synthesis in real time, allowing the reading of sequences up to tens of kilobases in length, with data formats including BAM and FASTQ. Oxford Nanopore MinION detects changes in electrical current as single DNA molecules pass through a nanopore, generating real-time sequence data up to several megabases long, with data formats including FAST5 and FASTQ.

The data processing workflow includes quality control (QC), sequence alignment, and variant detection. QC (e.g., using FastQC [18]) assesses the quality of the raw sequence data. Sequence alignment (e.g., using BWA or Bowtie2 [19]) maps the short-read sequences to a reference genome, producing alignment files in BAM format. Variant detection (e.g., GATK, Bayes, or Neural Network approaches [20]) identifies and annotates single-nucleotide variants (SNVs), insertions/deletions (Indels), and structural variants (SVs) in the genome. Furthermore, data archiving and annotation are crucial steps. Data archiving involves storing the processed data in databases for subsequent analysis and sharing. Data annotation uses gene function databases (such as Ensembl and RefSeq) to annotate the functions of detected variants and predict their impact on gene function.

#### Features of genomic data

Genomic data encompass various types and formats, each with unique characteristics and applications (Table 1). DNA sequence data, typically stored in FASTA format, are used for genome assembly and annotation. Variant calling data (such as VCF format) record genomic variation information, which is useful for genotyping and individual genomics studies. Alignment data (often in BAM and SAM formats) store sequencing reads aligned to the reference genome and are suitable for genome alignment and variant detection. Annotation data (often in GFF and GTF formats) describe the locations and annotations of genes, transcripts, and other functional elements in the genome. SV data (often in BED format) annotate large-scale structural variations in the genome. Methylation data (often in BED and BAM formats) reflect the DNA methylation status and are used in epigenetic studies. Copy number variation data (often in SEG and VCF formats) describe changes in the copy number of specific genomic regions and are commonly used in cancer research and genetic disorder studies. Genome-wide association study data (such as PLINK and VCF formats) identify genetic variants associated with specific traits. Single-cell genomic data (often in FASTQ, BAM, and VCF formats) provide genomic sequences and variation information for individual cells, revealing cellular heterogeneity. These data types and formats play crucial roles in genomic research, advancing the field of genome science.

**Table 1.** Various genomic data formats, characteristics, and example applications

Data type	Format	Characteristics	Example applications
DNA sequence data	FASTA	DNA sequences	Genome assembly, annotation
Variant call data	VCF	Genetic variants, functional annotations	Genotyping, population genetics
Alignment data	BAM, SAM	Sequence alignments	Genome alignment, variant detection
Annotation data	GFF, GTF	Gene annotations	Gene function study, transcript analysis
Structural variation data	BED	Locations of structural variations	Structural variant detection
Methylation data	BED, BAM	DNA methylation levels	Epigenetics studies
Copy number variation data	SEG, VCF	Copy number changes in the genome	Cancer research, genetic disorder studies
GWAS data	PLINK, VCF	Genetic variants associated with traits	Genetic association studies
Single-cell genomics data	FASTQ, BAM, VCF	Single-cell genome sequences and variants	Single-cell genomics research

### Transcriptomic data acquisition and parsing

Transcriptomics studies the expression of genes under specific conditions and at specific times, primarily analyzing the expression levels and patterns of mRNAs. Transcriptomic data are obtained through high-throughput sequencing technologies such as RNA sequencing (RNA-seq), single-cell RNA-seq (scRNA-seq), small RNA-seq, and spatial transcriptomics (ST). RNA-seq technology involves sequencing platforms such as Illumina HiSeq and Illumina NovaSeq. Illumina HiSeq and NovaSeq are known for their high throughput and high accuracy, with each sample typically generating data ranging from tens of megabytes to several gigabytes in formats such as FASTQ, BAM, and SAM. RNA-seq technology sequences cDNA copies of mRNA molecules to generate high-throughput short-read sequences, revealing gene expression profiles under different conditions.

scRNA-seq technologies, such as 10x Genomics Chromium, utilize microfluidics to independently process and sequence individual cells, providing gene expression information at the single-cell level. The data generated by scRNA-seq typically range from hundreds of megabytes to tens of gigabytes, in formats including FASTQ, BAM, and CSV. This technology can dissect the cellular heterogeneity in complex tissues and organs, revealing the diversity of cell types and states.

Small RNA-seq is an advanced high-throughput sequencing technique specifically designed for the analysis of small RNA molecules, typically from 18 to 30 nucleotides in length. These small RNAs encompass a variety of important regulatory molecules, including microRNAs (miRNAs), small interfering RNAs (siRNAs), piwi-interacting RNAs (piRNAs), and other small noncoding RNAs [21], all of which play fundamental roles in gene regulation, development, and cellular processes. By leveraging the sequencing platform, small RNA-seq enables the precise detection of expression levels and differential expression patterns of both established and novel miRNAs [22]. When integrated with transcriptome sequencing data obtained from the same biological sample, this methodology facilitates comprehensive analysis of miRNA expression alongside its target genes, thereby offering a robust investigative tool for elucidating the functional roles and regulatory mechanisms of RNA molecules.

ST is a technique that resolves RNA-seq data at high spatial resolution, enabling the profiling of all mRNAs in a single tissue section. This allows the localization and differentiation of actively expressed genes in specific tissue regions [23,24], suggesting promising applications in areas such as cancer, immunity, tumor–immune interactions, the tissue microenvironment, neurology, and development. 10X Visium is an ST technology used to study the gene expression patterns of tissues and cells in their native spatial context [25]. This technology allows researchers to simultaneously obtain expression information for thousands of genes within a tissue and relate it to the spatial architecture of the tissue.

The data processing pipeline includes QC, sequence alignment, and expression quantification. QC, e.g., using FastQC, assesses the quality of raw sequence data. Sequence alignment, e.g., using STAR or HISAT2 [26], aligns short-read sequences to a reference genome, producing alignment files in BAM format. Expression quantification, e.g., using TPMCalculator or HTSeq [27], measures gene expression levels, generating a gene expression count matrix. Additionally, data archiving and annotation are critical steps. Data archiving involves storing processed data in databases such as the National Center for Biotechnology Information (NCBI) Sequence Read Archive (SRA) database for subsequent analysis and sharing. Data annotation uses gene function databases (e.g., Ensembl and RefSeq) to provide gene function annotations, analyze gene expression patterns, and identify differentially expressed genes (DEGs). Through these detailed data acquisition and processing steps, researchers can comprehensively understand dynamic gene expression changes, providing essential foundational data for biological research and disease mechanism studies.

Transcriptomic data encompass various types and formats, each with unique characteristics and applications (Table 2). RNA sequence data are typically stored in FASTQ format, which records sequencing reads and their quality information. Alignment data (often in BAM and SAM formats) document the alignment of sequencing reads to the reference transcriptome, making them suitable for gene expression quantification and transcript structure analysis. Gene expression quantification data [often in FPKM (fragments per kilobase of exon model per million mapped fragments) and TPM (transcripts per million) formats] represent the expression levels of genes or transcripts. Differential expression analysis data, such as output formats from limma, DESeq2, and edgeR [28], are used to identify differences in gene expression under different conditions. Single-cell transcriptomics data (often in FASTQ, BAM, and CSV formats) provide gene expression profiles of individual cells, revealing cellular heterogeneity and complex biological processes. Noncoding RNA data (such as GFF and FASTA formats) describe the sequences and annotations of noncoding RNAs and are used to study their roles in gene regulation. These data types and formats play critical roles in transcriptomics research, advancing the understanding of gene expression and regulatory mechanisms.

**Table 2.** Transcriptomic data formats, characteristics, and example applications

Data type	Format	Characteristics	Example applications
RNA sequence data	FASTQ	RNA sequences, quality scores	RNA sequencing, transcript identification
Alignment data	BAM, SAM	Sequence alignments to reference transcriptome	Gene expression quantification, transcript structure analysis
Gene expression quantification	FPKM, TPM	Expression levels of genes or transcripts	Comparative gene expression studies
Differential expression analysis	Txt, CSV	Differential expression between conditions	Identification of differentially expressed genes
Single-cell transcriptomics data	FASTQ, BAM, CSV	Gene expression profiles at single-cell level	Single-cell heterogeneity analysis
Non-coding RNA data	GFF, FASTA	Sequences and annotations of noncoding RNAs	Studying gene regulation roles

### Acquisition and parsing of proteomic data

Proteomic research focuses on the expression, function, and interactions of proteins, which is essential for understanding their cellular functions and disease mechanisms. Proteomic data are obtained through various experimental techniques and advanced instruments, including MS and 2D gel electrophoresis. These techniques provide high-resolution and high-throughput protein identification and quantification data.

MS analysis is the core technology used in proteomics research, and the most commonly used instruments include the Thermo Fisher Orbitrap and Bruker timsTOF. The Thermo Fisher Orbitrap is known for its high resolution and sensitivity, generating data typically ranging from hundreds of megabytes to gigabytes per sample. MS analysis involves ionization, m/z analysis, and the generation of mass spectra, enabling the efficient identification and quantification of proteins in complex biological samples. 2D gel electrophoresis is a traditional protein separation technique that uses isoelectric focusing and sodium dodecyl sulfate–polyacrylamide gel electrophoresis for protein separation. Despite its lower resolution, 2D gel electrophoresis remains an important method for the initial separation and analysis of proteins and is particularly suitable for the preliminary separation and analysis of complex samples.

The data processing steps include QC, peptide matching, and quantitative analysis. MS data processing (using software such as MaxQuant or Proteome Discoverer) involves peak extraction, peptide identification, and protein quantification. Peptide matching (using database search engines such as Mascot or Sequest) compares experimental MS data with theoretical spectra in databases to identify proteins. Quantitative analysis (using methods such as label-free quantification or TMT labeling) quantifies protein expression levels and generates a protein quantification matrix. Additionally, data archiving and annotation are important steps. Data archiving involves storing processed data in databases for subsequent analysis and sharing. Data annotation uses protein function databases (such as UniProt and Pfam) for the functional annotation of proteins and for analyzing protein interaction networks and functional pathways. Through these detailed data acquisition and processing steps, researchers can comprehensively understand protein expression and functional changes, providing important foundational data for biological research and disease mechanism studies.

Proteomic data include various types and formats, each with unique characteristics and applications (Table 3). Protein sequence data are typically stored in FASTA format and record the amino acid sequences of proteins. MS data (often in mzML and RAW formats) contain mass spectra of proteins or peptides and are used for identification and quantitative analysis. Protein identification data (often in PEPXML and MZID formats) store information on proteins and peptides identified through MS analysis [29]. Protein quantification data (often in SILAC, iTRAQ, and TMT formats) are used for the relative or absolute quantification of protein abundance. Protein interaction data (often in SAINT and MIST formats) describe the interaction networks between proteins, revealing protein functions and signaling pathways [30]. Protein modification data (often in PTM format) include information on the posttranslational modifications of proteins and are used to study the roles of these modifications in regulation and function. These data types and formats play crucial roles in proteomic research, advancing the understanding of protein functions, interactions, and regulatory mechanisms.

**Table 3.** Various proteomic data formats, characteristics, and example applications

Data type	Format	Characteristics	Example applications
Protein sequence data	FASTA	Amino acid sequences of proteins	Protein identification and characterization
Mass spectrometry data	mzML, RAW	Mass spectra of proteins or peptides	Protein and peptide identification
Protein identification data	PEPXML, MZID	Identified proteins and peptides from mass spectrometry	Confirming protein presence and modifications
Protein quantification data	SILAC, iTRAQ, TMT	Relative or absolute quantification of protein abundance	Comparative proteomics, biomarker discovery
Protein interaction data	SAINT, MIST	Protein–protein interaction networks	Studying protein functions and pathways
Protein modification data	PTM	Posttranslational modifications of proteins	Understanding protein regulation and function

### Acquisition and parsing of epigenomic data

Epigenomics studies the regulatory mechanisms of gene expression without involving changes in the DNA sequence. It mainly addresses DNA methylation, histone modifications, and the roles of noncoding RNAs. Epigenomic data are obtained through various experimental techniques and advanced instruments, primarily bisulfite sequencing and chromatin immunoprecipitation sequencing (ChIP-seq). These techniques can efficiently reveal a comprehensive view of epigenetic modifications across the genome.

Bisulfite sequencing is a primary technique for studying DNA methylation, with commonly used platforms including Illumina HiSeq and NovaSeq. Bisulfite sequencing works by treating DNA with bisulfite, converting unmethylated cytosines (C) to uracil (U) while methylated cytosines (5mC) remain unchanged. After sequencing, the conversion of C to T in the original sequence reflects the methylation status. The data generated for each sample typically amount to several gigabytes, with formats including FASTQ, BAM, and BED.

ChIP-seq is used to study the genomic distribution of histone modifications and DNA-binding proteins. Common platforms for this technique include Illumina HiSeq and NovaSeq. ChIP-seq uses specific antibodies to enrich particular histone modifications or DNA-binding proteins, followed by high-throughput sequencing to reveal the binding sites of these modifications or proteins across the genome. The data generated for each sample typically amount to several gigabytes, with formats including FASTQ, BAM, and BED.

The data processing steps include QC, sequence alignment, and modification site detection. QC (e.g., using FastQC) assesses the quality of raw sequence data. Sequence alignment (e.g., using Bismark for bisulfite sequencing or Bowtie2 for ChIP-seq [31]) aligns short-read sequences to a reference genome, generating alignment files in BAM format. Modification site detection (e.g., using Bismark for bisulfite sequencing or magnetic-activated cell sorting for ChIP-seq) identifies and annotates methylation sites or protein-binding sites in the genome. Data archiving and annotation are also important steps. Data archiving involves storing processed data in databases for subsequent analysis and sharing. Data annotation uses gene function databases (such as Ensembl and RefSeq) to functionally annotate modification sites, predicting the effects of these modifications on gene function. Through these detailed data acquisition and processing steps, researchers can comprehensively elucidate the dynamic changes in genomic epigenetic modifications, providing crucial foundational data for biological research and disease mechanism studies.

Epigenomic data include various types and formats, each with unique characteristics and applications (Table 4). DNA methylation data are typically stored in the BIS-BED or BAM format, which records information on DNA methylation sites in the genome. ChIP-seq data (often in BED and BAM formats) contain information on DNA fragments bound to specific proteins and are used for studying chromatin states and transcription factor-binding sites. Chromatin accessibility data [such as ATAC-seq (assay for transposase-accessible chromatin with high-throughput sequencing) data, usually stored in BED and BAM formats] reveal open chromatin regions, indicating areas of active genes. Histone modification data (such as ChIP-seq data) record specific modification sites on histones, investigating their roles in gene regulation. Hi-C data (typically stored in BED and matrix formats) are used to analyze the 3D structure of chromatin, revealing physical contacts between different parts of the genome. These data types and formats play crucial roles in epigenomic research, advancing the understanding of gene expression and regulatory mechanisms.

**Table 4.** Various epigenomic data formats, characteristics, and example applications

Data type	Format	Characteristics	Example applications
DNA methylation data	BIS-BED, BAM	DNA methylation sites in the genome	Epigenetic regulation, cancer research
Chromatin immunoprecipitation (ChIP-seq) data	BED, BAM	DNA fragments bound to specific proteins	Chromatin state, transcription factor binding
Chromatin accessibility data	BED, BAM	Open chromatin regions	Active gene regions, regulatory elements
Histone modification data	BED, BAM	Specific histone modification sites	Gene regulation, chromatin structure
Chromosome conformation capture (Hi-C) data	BED, Matrix	3D chromatin structure	Genome organization, gene regulation
RNA modification data	BED, BAM	Chemical modifications on RNA molecules	RNA regulation, posttranscriptional control

The chemical modification of RNA is a recently discovered epigenetic regulatory mechanism within cells that plays a pivotal role in numerous biological processes. With the identification of more than 150 types of RNA modifications [32], such as N6-methyladenosine (m6A), 5-methylcytosine (m5C), pseudouridine (ψ), 5-hydroxymethylcytosine (hm5C), N1-methyladenosine (m1A), N7-methylguanosine (m7G), and N6,2'-O-dimethyladenosine (m6Am), the importance of RNA modifications has become increasingly apparent. Among these, m6A is among the most prevalent RNA modifications.

To advance our understanding of m6A modifications, a range of innovative technologies have been employed (Table 5), including dot-blot [33], high-performance liquid chromatography–tandem MS (HPLC-MS/MS) [34], methylated RNA immunoprecipitation sequencing (MeRIP/m6A-seq) [35], m6A-seq2 [36], m6A individual-nucleotide-resolution cross-linking and immunoprecipitation (MiCLIP/m6A-CLIP) [37], MAZTER-seq [38], m6A-selective allyl chemical labeling and sequencing (m6A-SAC-seq) [39], deamination adjacent to RNA modification targets (DART-seq) [40], nanopore RNA-seq [41], MeRIP–RT-qPCR (reverse transcription quantitative polymerase chain reaction) [42], methylate-sensitive endonuclease activity of MazF and the simultaneous amplification and testing (m6A-MazF-SAT) [43], picogram-scale m6A RNA immunoprecipitation and sequencing (picoMeRIP-seq) [44], and m6A-CT/single-nucleus m6A-cleavage under targets and tagmentation (sn-m6A-CT) [45]. Each of these methods has unique applications and characteristics. For example, m6A/sn-m6A-CT technology has been specifically tailored for single-cell m6A modification research. By employing the CT method, this technology effectively enriches m6A-modified RNA without requiring specific in vitro assay conditions, such as high temperature and alkalinity.

**Table 5.** Methods for identifying m6A modifications

Methods	Categories	Characteristics	References
Dot-blot	Detecting total m6A levels	The margin of error is substantial, difficult to precisely determine the modification of a specific RNA	[33]
HPLC-MS/MS	Detecting total m6A levels	Determining the sequence positions of modifications on RNA fragments	[34]
MeRIP/m6A-seq	Antibody dependent, high-throughput m6A sequencing	High-throughput, fast and cost-effective, identifies only regions of m6A hypermethylation	[35]
m6A-seq2	Antibody dependent, high-throughput m6A sequencing	Reduces technical replicate errors and library preparation costs, requires a large amount of RNA, high experimental costs	[36]
MiCLIP/m6A-CLIP	Antibody dependent, high-throughput m6A sequencing	Improve resolution, the binding efficiency of the antibody to RNA and the crosslinking efficiency can affect the final sequencing results	[37]
MAZTER-seq	Antibody-independent, high-throughput m6A sequencing	Cannot cover all possible m6A sites	[38]
m6A-SAC-seq	Antibody-independent, high-throughput m6A sequencing	Reaction based on enzymes may exhibit sequence specificity	[39]
DART-seq	Antibody-independent, high-throughput m6A sequencing	Easy to operate, highly specific, and relatively low amount of RNA needed	[40]
Nanopore RNA-sequencing	Antibody-independent, high-throughput m6A sequencing	Single-base level, costly, low accuracy, requires high RNA quality and input quantity	[41]
MeRIP–RT-qPCR	Detecting m6A modification of individual genes	Simple and practical, unable to provide single-base resolution	[46]
m6A-MazF-SAT	Detecting m6A modification of individual genes	Simple, user-friendly, provides rapid results, low false-positive rates, good reproducibility	[43]
picoMeRIP-seq	Detecting m6A modification of single cells	Applicable to low RNA/cell input amounts and suitable for single-cell MeRIP-seq	[44]
m6A-CT/sn-m6A-CT	Detecting m6A modification of single cells	Relies on a stringent in vitro assay	[45]

### Acquisition and parsing of metabolomic data

Metabolomics studies the composition of and changes in metabolites, primarily including qualitative and quantitative analyses of metabolites. Metabolomic data are obtained through techniques such as MS and NMR, which can reveal the dynamic regulation of metabolic pathways and interactions between metabolites within an organism.

MS is one of the primary techniques in epigenetic metabolomics research, with commonly used platforms including liquid chromatography–MS (LC-MS) and gas chromatography–MS (GC-MS). LC-MS technology, which combines LC separation and MS analysis, can efficiently identify and quantify metabolites in complex mixtures. Common platforms include the Thermo Fisher Orbitrap and Agilent Q-TOF. The data generated for each sample typically range from several hundred megabytes to several gigabytes, with formats including RAW, mzML, and mzXML.

NMR technology measures the signals of nuclear spins in samples on the basis of the principles of NMR and is used for analyzing unlabeled metabolites such as organic acids and small-molecule metabolites. NMR data typically have high structural information and low quantitative precision, but NMR offers unique advantages in the qualitative analysis and structural identification of metabolites. The data acquisition process includes several key steps. The first step is sample processing and extraction. Samples (such as tissues, cells, or biological fluids) need to undergo appropriate preparation and extraction to ensure the comprehensive extraction and stability of metabolites. Different sample types may require different extraction methods, such as the chloroform–methanol extraction method or the amino acid precipitation method.

The next step is the selection of the analytical platform and method optimization. For LC-MS, chromatographic separation and MS analysis must be optimized to improve the detection sensitivity and resolution of metabolites. For NMR, the spectral data acquisition and processing must be optimized to obtain clear and reliable NMR spectra.

In the sequencing step, LC-MS or NMR technology is used for high-throughput analysis of metabolites. LC-MS generates mass spectra of metabolites through efficient chromatographic separation and high-sensitivity MS detection. NMR measures the nuclear spin signals of metabolites, providing qualitative analysis of metabolite structure and composition. Finally, bioinformatics tools are used for data processing, metabolite identification, and quantitative analysis. The data processing steps include QC, peak extraction, and metabolite quantification. MS data processing, using tools such as XCMS or MzMine, involves mass peak extraction, metabolite identification, and quantification. NMR data (Table 6) processing, using tools such as Chenomx or Bruker TopSpin [47], involves NMR peak fitting and metabolite quantification. Additionally, data archiving and annotation are important steps. Data archiving involves storing processed data in databases for subsequent analysis and sharing. Data annotation uses metabolite databases, such as HMDB [48] and KEGG, for metabolite structure identification and functional annotation and for analyzing metabolic pathways and biomarkers. Through these detailed data acquisition and processing steps, researchers can comprehensively elucidate the composition and dynamic changes in metabolites in organisms, providing crucial foundational data for metabolic disease research and health management.

**Table 6.** Various metabolomic data formats, characteristics, and example applications

Data type	Data format	Characteristics	Example applications
Raw spectral data	RAW files	Native format from mass spectrometers, containing raw spectral data	Direct data analysis using vendor software
Raw spectral data	mzML	Open format for mass spectrometry data, containing raw spectral data and metadata	Standardized data sharing and analysis in proteomics workflows
Raw spectral data	mzXML	XML-based format for mass spectrometry data, similar to mzML	Data conversion and storage
Peak lists	mascot generic format (MGF)	Text-based format containing peak lists from MS/MS experiments	Database searching (e.g., Mascot and X! Tandem)
Identification results	mzldentML	Format for storing peptide and protein identifications	Data sharing and standardization of identification results
Identification and quantitation results	mzTab	Tab-delimited format for storing peptide and protein identification and quantitation	Data sharing and reporting in publications
Identification results	pepXML	XML-based format for storing peptide identification data	Data integration and pipeline processing
Identification results	protXML	XML-based format for storing protein identification data	Protein inference and result integration
Sequence data	FASTA	Text-based format for storing protein sequences	Database searching and sequence alignment
Submission format	PRIDE XML	Format used by PRIDE database for proteomics data submission	Data submission and sharing in PRIDE database

### Phenomic data

Phenomics studies the phenotypic characteristics of individuals, including their morphology, physiology, and behavior, and their relationships with genotype and environment. Data acquisition involves various techniques and methods, including imaging, physiological measurements, and biological and molecular analyses (Table 7). Imaging techniques are used to obtain the morphological structures and tissue characteristics of individuals. Imaging techniques commonly used in animals include MRI and CT, which are used to identify and quantify organ structures. Plant imaging techniques mainly involve high-resolution cameras and plant scanners to capture image data on root structures, leaf morphology, and flower structures. Drones can also be used to take photographs to collect data on plant growth in the field.

**Table 7.** Various phenomic data formats, characteristics, and example applications

Data type	Data format	Characteristics	Example applications
Image data	TIFF	High-quality raster graphics format, widely used in scientific imaging	Microscopy, high-throughput phenotyping
Image data	JPEG/PNG	Compressed image formats, commonly used for storing and sharing images	General image storage, web sharing
Image data	DICOM	Standard for medical imaging data, includes metadata	Medical imaging, MRI, CT scans
3D image data	NlfTl	Standard format for neuroimaging data, supports 3D/4D images	MRI, brain imaging
3D image data	OBJ/STL	Formats for storing 3D models	3D modeling, printing, and visualization
Time-series data	HDF5	Hierarchical data format, supports large, complex datasets	Multidimensional time-series data analysis, storage
Time-series data	CSV/TSV	Simple text formats for tabular data, easy to read and write	Storing and analyzing time-series data, simple data interchange
Morphological data	CSV/TSV	Simple text formats for tabular data, easy to read and write	Storing morphological measurements and annotations
Geospatial data	GeoTIFF	TIFF format with embedded georeferencing information	Remote sensing, GIS applications
Geospatial data	Shapefile	Vector format for geographic information system (GIS) data	Storing vector data for GIS applications
Genotypic–phenotypic data	VCF	Variant call format, stores gene variants with phenotypic associations	Genome-wide association studies (GWAS), genetic research
Phenotypic data	ISA-Tab	Tab-delimited format for representing experimental metadata	Multiomics and phenotypic data integration

The analysis of imaging data uses image processing software, such as ImageJ [49] and PlantCV [50], to extract and quantify morphological traits. Physiological measurements cover the physiological function parameters of organisms, such as blood pressure, heart rate, chlorophyll content, and photosynthesis rate. These parameters are monitored and recorded in real time via specialized equipment (such as blood pressure monitors, electrocardiographs, photosynthesis meters, and soil moisture sensors) to assess the physiological state and growth responses of individuals. In the data acquisition process, researchers must select appropriate experimental designs and techniques on the basis of the research subjects and scientific questions to ensure data accuracy and comparability. The optimization of these techniques includes sample preparation, standardization of measurement conditions, and selection of data processing and analysis methods. Ultimately, through these detailed data acquisition and processing steps, researchers can comprehensively elucidate the morphological structure, physiological functions, and molecular mechanisms of individuals, providing crucial foundational data for research on the genetic improvement, environmental adaptability, and disease resistance of both animals and plants.

### Single-cell omics

The acquisition and parsing of single-cell omics data have undergone important advancements in recent years (Table 8), driven by the need to understand cellular heterogeneity and dynamic biological processes at unprecedented resolution. High-throughput single-cell isolation technologies, such as microfluidics and fluorescence-activated cell sorting (FACS) [51], have enabled the precise manipulation and separation of individual cells. Techniques such as droplet-based microfluidics, exemplified by Drop-seq and inDrop, encapsulate single cells in droplets for high-throughput scRNA-seq. These innovations facilitate the processing of thousands of cells simultaneously, offering deep insights into the gene expression profiles of individual cells.

**Table 8.** Various single-cell data formats, characteristics, and example applications

Data type	Data format	Characteristics	Example applications
Single-cell RNA sequencing (scRNA-seq)	FASTQ, BAM, H5AD	High-resolution, captures transcriptomes of individual cells; high-throughput; potential for droplet-based and plate-based methods	Identifying cell types and states, studying cell differentiation and function
Single-cell ATAC sequencing (scATAC-seq)	FASTQ, BAM, H5AD	Measures chromatin accessibility at single-cell resolution; high-throughput; integrates with scRNA-seq data	Mapping regulatory elements, studying epigenetic changes and gene regulation
Single-cell DNA sequencing (scDNA-seq)	FASTQ, BAM, VCF	Captures genomic variations at single-cell resolution; identifies mutations and copy number variations	Analyzing genetic heterogeneity in tumors, tracking clonal evolution
Single-cell DNA methylation sequencing	FASTQ, BAM, BED	Measures DNA methylation patterns in single cells; provides epigenetic profiles	Studying epigenetic regulation, cell lineage tracing, disease mechanisms
Single-cell proteomics (cyTOF)	FCS, CSV, H5AD	Measures protein expression at single-cell resolution using mass cytometry, high-throughput	Profiling immune cell populations, studying protein expression dynamics
Single-cell multiomics (e.g., sci-CAR and Paired-Seq)	FASTQ, BAM, H5AD	Simultaneously profiles multiple omics layers (e.g., transcriptome and epigenome) in single cells	Integrating transcriptomic and epigenomic data, understanding gene regulation
Spatial transcriptomics	FASTQ, BAM, H5AD, GFF3	Combines high-throughput sequencing with spatial information; captures gene expression in tissue context	Studying spatial gene expression patterns, tissue organization
Single-cell RNA velocity	FASTQ, BAM, H5AD	Infers dynamic changes in RNA expression over time; uses spliced and unspliced RNA reads	Elucidating cell state transitions, studying developmental processes

Advancements in sequencing technologies have further propelled single-cell omics. NGS platforms have been optimized for single-cell applications, providing high sensitivity and throughput. Technologies such as 10x Genomics Chromium and Smart-seq3 allow the robust sequencing of thousands of individual cells in parallel, revealing intricate details of cellular states. Additionally, ST techniques, such as Slide-seq and 10x Genomics Visium [25], combine high-throughput sequencing with spatial information, enabling the localization of gene expression within tissue sections. These methods provide a spatial context for single-cell data, enhancing our understanding of cellular interactions and tissue organization.

The parsing of single-cell omics data has also seen remarkable progress. Advanced bioinformatics tools have improved data preprocessing, ensuring high-quality datasets for downstream analysis. Tools such as Cell Ranger (10x Genomics) [53] and STARSolo [54] facilitate the alignment and quantification of single-cell RNA-seq data, whereas QC tools such as Seurat and Scrublet [55] identify and remove low-quality cells and doublets. These preprocessing steps are crucial for maintaining data integrity and reliability. Dimensionality reduction techniques, including principal components analysis (PCA), uniform manifold approximation and projection (UMAP), and t-distributed stochastic neighbor embedding (t-SNE), simplify high-dimensional data, making them easier to visualize and interpret. Clustering algorithms such as those of Louvain and Leiden identify distinct cell populations on the basis of gene expression profiles, enabling the discovery of new cell types and states.

The integration of multiomics data has become increasingly sophisticated, with tools such as Seurat [56], Harmony [57], and LIGER [58] addressing batch effects and allowing comparative analyses across conditions. Latent space embedding methods, such as multiomics factor analysis (MOFA) [59] and variational autoencoders (VAEs) [60], embed multiomics data into a common latent space, uncovering shared and unique features across different omics layers. These approaches facilitate a holistic understanding of cellular functions and interactions. Trajectory inference and dynamic modeling tools, including Monocle3 [61], Slingshot [62], and PAGA [63], reconstruct developmental trajectories and lineage relationships, providing insights into cellular differentiation and dynamic processes. Additionally, methods such as scVelo [64] and RNA velocity [65] infer cell state transitions and dynamic changes over time, enhancing our understanding of cellular dynamics.

Functional annotation and pathway analysis are essential for interpreting single-cell omics data. Gene set enrichment analysis tools, such as GSEA [66] and enrichR [67], identify enriched pathways and biological processes, facilitating the functional annotation of single-cell clusters. Regulatory network inference tools, such as SCENIC [68] and CellOracle [69], reconstruct gene regulatory networks from single-cell data, shedding light on the regulatory mechanisms driving cellular behaviors. These advances collectively enable a deeper and more comprehensive understanding of cellular heterogeneity and dynamics, paving the way for new discoveries in biology and medicine. The integration of high-throughput acquisition technologies and advanced computational tools has transformed single-cell omics, providing unprecedented insights into the complexities of biological systems.

## Multiomics Data Association Analysis

### Features of multiomics data

Each type of data format corresponds to specific measurement techniques and technologies used in omics research, each with its own data handling and analysis requirements. Integrating these diverse data formats is crucial for obtaining comprehensive biological insights and understanding complex biological systems (Table 9).

**Table 9.** Characteristics of multiomics data

Characteristics	Description	Example
High-dimensional data	Datasets with a large number of variables (features) and relatively few observations (samples), common in omics studies	Gene expression datasets with thousands of genes measured across a few dozen samples
Heterogeneity	Variability and diversity within biological data, reflecting differences between individuals, tissues, or conditions	Differences in gene expression profiles between healthy and diseased tissue samples
Sparsity	The presence of many zero or near-zero values in the dataset, indicating that many features are not expressed or detected in all samples	Metabolomics data where many metabolites are below the detection limit in some samples
Noise	Random variations and measurement errors that obscure the true signal in the data, requiring robust preprocessing and analysis methods	Technical variations in sequencing data leading to fluctuations in read counts
Batch effects	Systematic nonbiological differences introduced during data generation, often due to variations in sample processing or equipment	Variations in gene expression data due to different sequencing runs or labs
Missing data	Incomplete datasets where some features or samples have not been measured or have missing values, complicating analysis and interpretation	Missing proteomics data for certain proteins in some samples due to detection limits
Data integration	Combining data from multiple omics platforms (e.g., genomics, transcriptomics, and proteomics) to obtain a comprehensive view of biological systems	Integrating RNA-seq gene expression data with proteomics data to correlate mRNA levels with protein abundance
Multicollinearity	The presence of high correlations among some features, which can complicate statistical analyses and model interpretation	High correlation between expression levels of coregulated genes in a gene expression dataset
Dimensionality reduction	Techniques used to reduce the number of variables under consideration, making analysis more tractable and highlighting key patterns	Principal components analysis (PCA) applied to metabolomics data to identify key metabolic pathways
Feature selection	Identifying and selecting the most relevant features for analysis, improving model performance and interpretability	Using statistical tests to select differentially expressed genes for further analysis in transcriptomic research
Temporal dynamics	Changes in biological data over time, important for understanding dynamic processes such as development or response to treatment	Time-course gene expression data tracking changes in mRNA levels after drug treatment
Interomics relationships	Associations and interactions between different types of omics data, revealing complex regulatory and functional networks	Correlating DNA methylation levels with gene expression data to study epigenetic regulation of gene activity

Genomic data are typically represented as sequences of nucleotides (e.g., A, T, C, and G) or single-nucleotide polymorphisms (SNPs). Transcriptomic data include gene expression levels (continuous) or counts of transcripts (discrete). Proteomic data quantify protein expression levels (continuous) or the presence/absence of proteins (binary). Metabolomic data measure concentrations of metabolites (continuous or discrete) in biological samples. Phenotypic data cover clinical traits, demographic information, and other non-omics data relevant to the study.

### Multiomics data integration algorithms

#### Data preprocessing

**Fig. 2.**
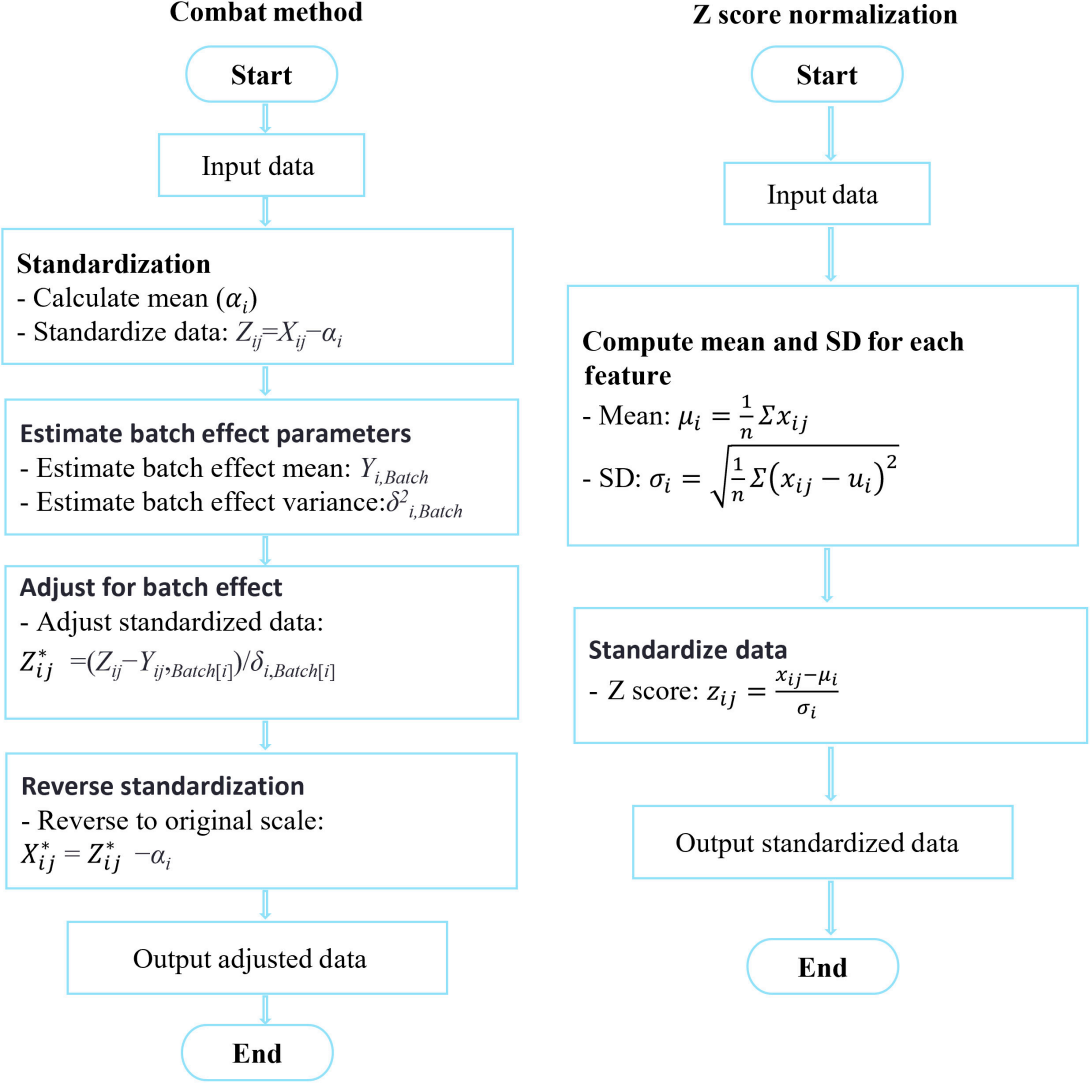
The mathematical formulation of the 2 methods and their data preprocessing pipeline. Symbols in the ComBat method: *Y_ij_* represents the observed data value at row *i* and column *j*.

The main methods in omics data preprocessing are normalization and batch effect correction (Fig. 2). Normalization ensures that different data types and scales are comparable. Common methods include *Z* score normalization, which transforms the data to have a mean of 0 and an SD of 1, and min–max normalization, which scales the data to a fixed range, usually [0, 1] [70]. Batch effect correction addresses nonbiological variations caused by differences in experimental conditions or batch processing. ComBat [71] is a widely used method that employs an empirical Bayes approach to adjust batch effects in high-dimensional data. These techniques are crucial for ensuring data comparability and integrity in omics studies.

##### Normalization

Normalization ensures that different data types and scales are comparable. Common normalization methods include *Z* score normalization, which transforms the data to have a mean of 0 and an SD of 1, and min–max normalization, which scales the data to a fixed range, usually [0, 1].

##### Batch effect correction

Batch effects are nonbiological variations caused by differences in experimental conditions or batch processing. Different omics data may have varying levels of noise and missing data, necessitating cleaning and imputation. For missing data imputation, researchers often use various methods to handle incomplete datasets. Common methods include ComBat, *k*-nearest neighbors (KNN), multiple imputation by chained equations (MICE) [72], and matrix factorization techniques.

ComBat: An empirical Bayes method, which is widely used in genomics and other omics studies, is used to adjust for batch effects in high-dimensional data.

KNN: KNN is a supervised learning algorithm used for classification and regression tasks, which operates by identifying the *K* closest training samples to a given input on the basis of a distance metric, typically the Euclidean distance. The Euclidean distance between 2 samples $x_{i}=(x_{i1},x_{i2},\ldots ,x_{\text{in}}\;)$ and $x_{j}=(x_{j1},x_{j2},$$\ldots ,x_{\mathrm{jn}})$ is calculated as $d\left(x_{i},\;x_{j}\right)=\sqrt{\sum_{k=1}^{n}{\left(x_{\mathrm{ik}}-x_{\mathrm{jk}}\right)}^{2}}$. For classification, KNN predicts the class of a given input by majority voting among its *K* nearest neighbors, whereas for regression, it predicts the value by averaging the values of the *K* nearest neighbors.

MICE: MICE is a method for handling missing data by iteratively imputing missing values for each variable via a regression model, where each variable with missing values is modeled to be conditional on the other variables. Initially, missing values are imputed via simple methods, such as mean imputation. Then, for each variable *Y_j_* with missing values, a regression model $Y_{j}=\beta_{0}+\beta_{1}X_{1}+\beta_{2}X_{2}+\ldots +\beta_{p}X_{p}+\epsilon$ is fitted with the other variables *X_-j_* as predictors. The missing values are imputed on the basis of this model, adding random error to reflect uncertainty. This process is repeated multiple times, creating *m* complete datasets. The overall estimate is calculated as the average of the estimates from these datasets: $\bar{Q}=\frac{1}{m}\sum_{k=1}^{m}Q_{k}$. The overall variance is given by $T=\bar{U}+\left(1+\frac{1}{m}\right)B$, where $\;\bar{U}=\frac{1}{m}\sum_{k=1}^{m}U_{k}$ is the within-imputation variance and $B=\frac{1}{m-1}\sum_{k=1}^{m}{\left(Q_{k}-\bar{Q}\right)}^{2}$ is the between-imputation variance.

##### Denoising

Commonly employed denoising methods include matrix factorization techniques such as PCA [73] and singular value decomposition (SVD) [74]. These approaches address missing values or reduce dataset dimensionality by projecting the data into a lower-dimensional space.

Matrix factorization techniques: Researchers often utilize matrix factorization techniques, such as SVD and nonnegative matrix factorization (NMF) [75], to reconstruct incomplete datasets. In SVD, the observed data matrix *X* is decomposed into $X=U\Sigma V^{T}$, where *U* and *V* are orthogonal matrices, and Σ is a diagonal matrix of singular values. For missing data, an iterative approach is used in which initial guesses for missing values are made, followed by repeated SVD decompositions to refine these estimates until convergence. NMF, on the other hand, approximates *X* by the product of 2 nonnegative matrices, *W* and *H*, such that *X* ≈ *WH*, and minimizes the reconstruction error via the Frobenius norm: $\min_{W,H}{\left\|X-\mathrm{WH}\right\|}_{F}^{2}$. These methods leverage the underlying low-rank structure of the data to effectively fill in missing values, providing a robust approach to handle incomplete datasets.

PCA: PCA is particularly advantageous for diminishing data complexity and noise, thereby enhancing model predictive performance, although it may result in the loss of some information from the original data.

SVD: Conversely, SVD is suitable for managing large-scale datasets and nonlinear data, offering more effective handling of missing values. However, it also imposes certain assumptions regarding data types and distributions, which may limit its applicability.

##### Data QC software

Bioinformatics data QC software plays a crucial role in ensuring the accuracy and reliability of biological data analysis. Some of the most widely used tools for this purpose are Trimmomatic [76], fastp [77], FastQC [78], SOAPnuke [79], and LongQC [80] (Table 10). Long-read sequencing technologies, such as SMRT sequencing by PacBio, nanopore sequencing by ONT, synthetic long reads (SLRs), and linked-read sequencing, rely on different QC principles, and the corresponding tools are also different. For example, FastQC, a widely acclaimed tool, plays a crucial role in evaluating the quality of sequencing data. It produces comprehensive reports that encompass a range of quality metrics, including per-base sequence quality, sequence length distribution, GC content, and sequence duplication levels. By utilizing these metrics, FastQC aids researchers in detecting potential issues such as sequencing errors, adapter contamination, or substandard data quality.

**Table 10.** Tools for sequence quality control

Tools	Characteristics	Technologies	References
Trimmomatic	Flexible and exhaustive functions	Short reads, SLR, and linked reads	[76]
fastp	Ultrafast; exhaustive functions	Short reads, SLR, and linked reads	[77]
FastQC	Excellent visualization	Short reads, SLR, and linked reads	[78]
SOAPnuke	Reduced memory; predefined modules	Short reads, SLR, and linked reads	[79]
LongQC	Computationally efficient and user-friendly	Long reads	[80]

#### Integration of multiomics data

Multiomics data usually come from different experimental platforms with varying data structures and distributions, requiring effective integration. iCluster (iClusterPlus, iClusterBayes) [81] and MOFA [59] are 2 commonly used methods for multiomics data analysis (Table 11) and are designed to integrate and process various types of omics data, such as genomics, transcriptomics, and epigenomics data. iCluster integrates data within a Bayesian framework, first standardizing different types of omics data, then uses Bayesian variable selection methods to jointly model latent variables, and finally, sample subgroups with similar molecular characteristics are identified through clustering algorithms. This method is particularly suitable for sample clustering analysis and molecular subtype identification.

**Table 11.** The methods for multiomics data analysis

Aspect	iCluster	MOFA
Application	Integrative clustering of multiomics data	Multiomics factor analysis (MOFA)
	Identification of clusters/patterns across different omics layers	Dimensionality reduction and factor analysis
	Suitable for datasets with discrete clusters	Suitable for datasets with continuous and discrete variables
Data type	Discrete and categorical omics data	Continuous and discrete omics data
Input	Multiomics datasets (e.g., genomics and transcriptomics)	Multiomics datasets (e.g., genomics and transcriptomics)
Methodology	Clustering algorithms (e.g., *k*-means and hierarchical clustering)	Matrix factorization methods (e.g., PCA and NMF)
	Integration of omics layers using similarity measures	Incorporation of prior knowledge (e.g., pathway information)
Output	Cluster assignments for samples	Latent factors explaining variation in data
	Visualization of integrated clusters	Visualization of factor loadings and latent variables
Advantages	Explicitly handles discrete data types	Handles both continuous and discrete variables seamlessly
	Provides interpretable clusters	Captures complex relationships across omics data
	Robust to noise and missing data	Allows incorporation of prior knowledge
Disadvantages	May oversimplify continuous data	Complexity in interpretation of latent factors
	Limited to predefined clustering algorithms	Sensitivity to model assumptions and hyperparameters
Software tools	iClusterPlus, iClusterBayes	MOFA (R package), MOFA2

MOFA, on the other hand, is based on a factor analysis model that assumes that observed data can be represented as a linear combination of latent factors, thereby revealing the common structure among multiple types of omics data. The MOFA estimates model parameters via the expectation–maximization (EM) algorithm [82] and variational inference [83], ensuring stability and interpretability when handling high-dimensional data. By analyzing latent factors, MOFA helps understand the relationships between different types of omics data and reveals underlying biological processes, such as cell states and molecular pathways.

In summary, iCluster and MOFA each have strengths in handling multiomics data. iCluster is well suited for sample clustering and molecular subtype identification through joint modeling of latent variables, whereas MOFA reveals common biological processes across multiple types of omics data via a factor analysis model. Researchers can choose the most appropriate method on the basis of their specific research goals and data characteristics.

#### Pattern recognition and feature extraction

Pattern recognition and feature extraction play crucial roles in identifying key patterns and features in high-dimensional data, thereby facilitating the understanding of underlying biological mechanisms. Various algorithms and methods are employed in this domain to enhance data analysis and interpretation. Dimensionality reduction and feature selection techniques, such as LASSO [84], elastic net [85], PCA, t-SNE [86], and UMAP [87], are instrumental in identifying important features by reducing the complexity of the data while retaining essential information. Clustering analysis methods, including *k*-means [88], hierarchical clustering [89], and DBSCAN [90], are utilized to uncover natural groupings within the data, further aiding in the discovery of meaningful patterns and insights. These combined approaches enable researchers to effectively process and interpret complex biological data, leading to a deeper understanding of biological processes and systems.

#### Data integration and association analysis

Data integration and association analysis are essential for identifying the correlations and causal relationships between different types of omics data. To achieve this, various algorithms and methods are utilized. Correlation analysis techniques, such as the Pearson correlation coefficient and Spearman correlation coefficient, are employed to assess the linear or nonlinear relationships between different omics datasets. Network analysis tools, such as Cytoscape [91], are used to construct and analyze interaction networks among genes, proteins, and metabolites to obtain insights into the complex interplay within biological systems. Additionally, multivariate analysis methods, including partial least squares (PLS) regression [92] and canonical correlation analysis (CCA) [93], are applied to identify underlying associations across multiple omics datasets. These approaches collectively enable researchers to integrate and interpret diverse biological data comprehensively, uncovering the intricate relationships and mechanisms that drive biological processes.

#### Biological interpretation and validation

Biological interpretation and validation are crucial steps in transforming algorithmic results into meaningful biological knowledge, which is then subject to experimental confirmation. This process involves several methods and strategies. Functional enrichment analyses, such as GSEA [66] and metabolite set enrichment analysis (MSEA) [94], are employed to elucidate the biological functions of genes, metabolites, and proteins identified by algorithms. Pathway analysis tools, including KEGG [95] and Reactome [96], assist in understanding how specific pathways are represented and behave across different omics datasets. Additionally, experimental validation is conducted to confirm the algorithmic predictions by verifying the roles of key genes, proteins, or metabolites through laboratory experiments. These approaches ensure that computational predictions are accurately interpreted within a biological context and validated through empirical evidence, bridging the gap between data analysis and biological discovery.

### R packages and software

iCluster, iClusterPlus [97], and iClusterBayes [98] are nonparametric tools designed for the integrative analysis of multiomics datasets. iClusterPlus, an R package, extends the original iCluster method by supporting a wider range of data types and clustering algorithm options, making it versatile for various integrative analyses. iClusterBayes, on the other hand, is a Bayesian variant of the iCluster method, also implemented in R, and is used for integrative clustering analysis of multiomics data with a probabilistic approach. Both tools are pivotal for uncovering complex biological insights by integrating diverse omics data (Table 12).

**Table 12.** Various methods implemented in R

Methods	R package
Matrix factorization methods	
Integrative nonnegative matrix factorization (intNMF)	intNMF
iClusterPlus	iClusterPlus
Multiple co-inertia analysis (MCIA)	Omicade4
Sparse generalized canonical correlation analysis (SGCCA)	RGCCA
Multiomics factor analysis (MOFA)	MOFAtools
Partial least squares (PLS)	Pls, caret, mixOmics, plsVarSel
Nonnegative matrix factorization (NMF)	NMF, nnmf, BiocGenerics, NMFEM
Linked inference of genomic experimental relationships (LIGER)	riger
Graph-based methods	
Similarity network fusion (SNF)	SNFtool
MoCluster	mosga
Cancer integration via multikernel learning and regularized manifold learning (CIMLR)	CIMLR
iGraph	igraph
Multiple canonical correlation analysis (MultiCCA)	PMA, CCA, RGCCA
Consensus clustering methods	
PINSPlus	PINSPlus
ConsensusClustering	Consensus, ClusteringPlus
Cluster of cluster assignments (COCA)	ConsensusClusterPlus
Other methods	
Regularized generalized canonical correlation analysis (RGCCA)	RGCCA
Low-rank approximation clustering (LRACluster)	LRACluster
Kernel	mixKernel
Data integration analysis for biomarker discovery using latent components (DIABLO)	mixOmics
Joint and individual variation explained (JIVE)	JIVE
Multiblock principal components analysis (MB-PCA)	mixOmics, BlockPCA
Structuration des tableaux a trois indices de la statistique (STATIS)	FactoMineR, ade4, mixOmics, tensorBSS
Integrative multiple correspondence analysis (IntMCA)	mixOmics, FactoMineR, ade

MetaboAnalyst [99] is a specialized platform for the analysis of metabolomics data and offers functionalities for data preprocessing, statistical analysis, and pathway enrichment analysis, facilitating comprehensive metabolomic studies. Galaxy is an open, web-based bioinformatics platform that supports the integration and analysis of multiple types of omics data, providing user-friendly data processing capabilities. KNIME is an open-source platform for data analytics, reporting, and integration that supports a wide variety of data formats and advanced analytical methods [100]. Cytoscape [91] is a widely used network visualization and analysis platform that integrates multiomics datasets and performs network analysis; it is often used in conjunction with plugins such as “NetworkAnalyzer” for enhanced functionality. These tools collectively increase the capacity for sophisticated data integration and analysis, enabling researchers to derive meaningful biological insights from complex datasets.

### Webservers

JBrowse is a versatile genome browser designed to display and interact with multiomics data [101]. It supports the visualization of a wide array of datasets, including genomic sequences, gene expression profiles, and DNA methylation patterns. By providing an intuitive interface, JBrowse allows researchers to easily navigate different types of omics data and gain comprehensive insights into genomic structures and regulatory mechanisms. This tool is particularly useful for integrating various omics layers, enabling the simultaneous examination of genetic, epigenetic, and transcriptomic information, which facilitates a holistic understanding of complex biological systems.

OmicsNet2.0 [102] is a network-based platform designed for the integrative analysis of multiomics data and supports the integration of proteomics, metabolomics, and genomics datasets. It enables the comprehensive exploration of biological interactions and pathways across different omics domains.

DR-omics [103] is a web-based platform dedicated to integrating and analyzing multiomics data that offers interactive networks and functional analyses to uncover relationships and biological insights within complex datasets.

TIMER [104] and CIBERSORT [105] are tools specifically designed for analyzing immunogenomic data and are commonly used in studies of the tumor microenvironment. They integrate gene expression data to provide insights into immune cell infiltration and immune response dynamics, facilitating research on immune-related aspects of diseases such as cancer (Table 13).

**Table 13.** Various web-based platforms for integrating and analyzing multiomics data

Webservers	Web link	Free of charge
OmicSoft	https://digitalinsights.qiagen.com/products-overview/discovery-insiqhts-portfolio/analysis-and-visualization/qiagen-omicsoft-suite/	No
HiPlot	https://hiplot.cn/	No
ImageGP	https://www.bic.ac.cn/lmageGP/	Yes
Majorbio Cloud	https://www.majorbio.com/	No
STOmicsCloud	https://www.stomics.tech/products/BioinfoTools/STOmicsCloud	No
Westlake Omics	https://www.westlakeomics.com/products/omic-cloud-platform/	No
OmicShare	https://www.omicshare.com/	No
BioLadder	https://www.bioladder.cn/web/#/pro/index	No
Metware Cloud	https://cloud.metware.cn/#/home	No
iOmics Cloud	https://iomicscloud.com/	No
ExpOmics	http://www.biomedical-web.com/expomics/home	Yes
Tencent HealthCar	https://cloud.tencent.com/product/omics	No
omicstudio	https://www.omicstudio.cn/home	No
OmicSolution	https://www.omicsolution.com/wkomics/wkold/	No
NovoMagic	https://magic-plus.novogene.com/#/	No
Dr. Tom	https://biosys.bqi.com/	No
BioDeep	https://www.biodeep.cn/home	No
OmicsAnalyst	https://www.omicsanalyst.ca/OmicsAnalyst/home.xhtml	Yes
Epigenetics Cloud	https://sinomics.com/CLOUD/	No
Jizhi Gene Database	https://omics.smartgenomics.net/#/home	Yes
APT-BioCloud	https://bio-cloud.aptbiotech.com/login	No
Sangon Biotech Cloud	https://ngs.sangon.com/	No
Wei ShengXin	https://www.bioinformatics.com.cn/	No
Wekemo Bioincloud	https://www.bioincloud.tech/	Yes
BioCloud	https://biocloud.sjtu.edu.cn/	No
Kaitai Cloud	https://kaitai.cloud/tools	No
Jingjie Cloud	http://114.115.141.182/#/auth/login	No
VAZYME	http://cloud.vazyme.com:83/	No
GENE	https://www.generover.com/#/index	No
Galaxy	https://useqalaxy.cn/	No
TianyiCloud	https://cloud.dftianyi.com/home/index	No
Oebiotech	https://cloud.oebiotech.com/#/home	No
BMKCLoud	https://www.biocloud.net/	No

### Database

The Gene Expression Omnibus (GEO), maintained by NCBI, is a public functional genomics data repository that stores a large amount of gene expression and high-throughput sequencing data, covering both transcriptomics and epigenomics [106]. ArrayExpress [107], which is maintained by EMBL-EBI, is a database of gene expression experimental data and supports the submission and retrieval of various omics data, with a focus on gene expression and transcriptomics. The Cancer Genome Atlas (TCGA) [108] is a large-scale collaborative project that collects multiomics data from various cancers, including cancer genomics, transcriptomics, epigenomics, proteomics, and clinical data, with the aim of studying the molecular mechanisms of cancer and discovering new therapeutic targets. The Encyclopedia of DNA Elements (ENCODE) project [109] is dedicated to identifying and annotating all functional elements, encompassing genomics, transcriptomics, and epigenomics, with a focus on transcription start sites, enhancers, and repressors. The Genotype-Tissue Expression (GTEx) project [110] studies gene expression and genetic variation across different tissues and organs, providing extensive multitissue gene expression data and covering both genomics and transcriptomics.

### Machine learning, deep learning, and large language models

Machine learning and deep learning techniques are instrumental in predicting biological processes and disease mechanisms by leveraging advanced algorithms. Supervised learning methods, such as random forests (RFs) [111], support vector machines (SVMs) [112], and deep neural networks (DNNs) [113], are employed for classification and regression tasks and provide powerful predictive capabilities. Unsupervised learning approaches, such as autoencoders [60] and GANs [114], are utilized for feature extraction and data generation, offering insights into the underlying structure of the data. Additionally, ensemble learning techniques, such as XGBoost [115] and LightGBM [116], are used to enhance model performance and stability, ensuring more accurate and reliable predictions. These diverse machine learning methodologies collectively advance the field of computational biology by enabling the precise modeling and understanding of complex biological systems (Table 14).

**Table 14.** The application of artificial intelligence technology in omics

Omics	Applications	References
Genomics	Machine-learning-based genomic selection, improving genome annotation using machine learning, deep learning models for CRISPR/Cas9 off-target cleavage prediction, machine learning for functional gene prediction	[120–127]
Transcriptomics	Gene expression inference with deep learning, deep learning of the tissue-regulated splicing, recurrent neural network for predicting transcription factor-binding sites	[128–131]
Proteomics	Identifying proteomic risk markers using deep learning, prediction of protein–peptide interactions and signaling networks using machine learning, predicting the sequence specificities of DNA- and RNA-binding proteins by deep learning, predicting protein structure with AlphaFold	[132–136]
Metabolomics	Deep learning for stratification of metabolic phenotypes, deep learning for predicting metabolic pathways	[137–141]
Single-cell omics	Transformer for cell type annotation prediction, using large language models for cell type classification and gene property prediction	[142,143]
Epigenomics	Deep learning approach to automate whole-genome prediction of diverse epigenomic modifications in plants	[144]

Recent advances in large-scale machine learning models have ushered in a new era of multiomics analysis, enabling unprecedented insights into complex biological systems. These innovations leverage the power of deep learning architectures to integrate and analyze diverse omics data, providing a comprehensive understanding of biological functions and disease mechanisms.

Originally developed for natural language processing, transformer models such as BERT [117] and GPT [118] have been adapted for multiomics data integration. These models excel at capturing long-range dependencies and can simultaneously process genomics, transcriptomics, metabolomics, and epigenomics data. This capability allows the discovery of intricate relationships and interactions across different biological layers.

Biological systems are inherently network-like, with complex interactions between genes, proteins, and metabolites. Graph neural networks (GNNs) are particularly well suited to model these interactions, facilitating the analysis of biological pathways and networks. By leveraging GNNs, researchers can uncover new insights into cellular processes and disease mechanisms.

Large models can integrate diverse data types, such as sequence data, expression profiles, and metabolic pathways, within a unified framework. This multimodal approach enhances the ability to predict phenotypic outcomes and understand the underlying biological mechanisms. Techniques such as VAEs [119] and other latent space models can be used to embed multiomics data into a common latent space. This approach allows the identification of shared features and patterns that are not apparent when each omics layer is analyzed independently.

Data standardization and interoperability are essential for effective multiomics analysis. Large models benefit from standardized data formats and comprehensive data repositories, enabling robust and reproducible analyses across different studies and datasets.

### Proposed solution: Multiview graph generative autoencoder network

The proposed algorithm, named the multiview graph generative autoencoder network (MV-GGAN), combines the strengths of multiview learning, graph convolutional networks (GCNs), GANs, and multimodal variational autoencoders (MVAEs) [145] to achieve comprehensive integration and feature extraction from multiomics data.

MV-GGAN begins by representing the multiomics data in different views, with each view corresponding to a distinct omics dataset, such as genomics, proteomics, or metabolomics. These diverse datasets are preprocessed through normalization and standardization techniques to ensure consistency and comparability. A multilayer graph structure is then constructed on the basis of the biological interactions between the different omics layers, forming a comprehensive gene–protein–metabolite network. This graph structure serves as the foundation for subsequent feature extraction.

In the feature extraction phase, GCNs are employed to learn the intricate relationships within the graph structure. GCNs are particularly effective in capturing the topological features of the graph and leveraging the connectivity information to generate robust node embeddings. These embeddings represent the features of genes, proteins, and metabolites within the multiomics network.

To address the issue of incomplete or missing data, GANs are integrated into the MV-GGAN framework. GANs consist of a generator and a discriminator that work in tandem to generate realistic data. The generator learns to produce plausible data points, whereas the discriminator aims to distinguish between real and generated data. Through this adversarial process, GANs enhance the overall data completeness and integrity, ensuring a more reliable dataset for downstream analysis.

The core innovation of MV-GGAN lies in the integration of MVAEs for multiview feature fusion and latent variable learning. MVAEs encode each omics view into a shared latent space, capturing the underlying distributions and relationships between the different datasets. By learning a common representation, MVAEs enable the fusion of multiomics data in a way that preserves the unique information from each view while also capturing their interdependencies. The latent variables extracted by MVAEs serve as a rich source of features for subsequent analyses.

The training process of MV-GGAN involves several stages. Initially, the GCNs are trained to extract node features from the graph structure. Concurrently, the GANs are trained to generate missing data points, enhancing data completeness. Finally, the MVAEs are trained to encode the multiview data into a shared latent space and decode them back to their original forms. This joint training ensures that the features learned are both comprehensive and representative of the underlying biological processes.

The MV-GGAN algorithm leverages the combined power of multiview learning, GCNs, GANs, and MVAEs to provide a novel solution for multiomics data integration and feature extraction. By addressing data heterogeneity and incompleteness while preserving unique and shared information from different omics layers, MV-GGAN offers a powerful tool for uncovering complex biological mechanisms and elucidating multiomics interactions. This innovative approach holds great promise for advancing multiomics research.

### Infrastructure requirements and challenges

Multidimensional omics data analysis requires substantial computational resources due to the complexity and volume of data involved [146]. Essential equipment includes high-performance computing (HPC) clusters featuring multicore processors such as Intel Xeon or AMD EPYC, which enable parallel processing and the efficient handling of large datasets. Additionally, high-end graphics processing units (GPUs) such as NVIDIA Tesla or AMD Radeon Pro can importantly accelerate machine learning algorithms and data processing tasks, especially for deep learning models. A large RAM capacity, typically 128 GB or more, is necessary to store and manipulate big data sets in memory, reducing the reliance on disk I/O operations and improving processing speed.

The storage needs for omics data analysis are extensive, often requiring terabytes (TB) of space to accommodate raw and processed data, such as sequencing reads, MS data, and imaging data. Compared with traditional hard disk drives (HDDs), fast I/O storage solutions, including solid-state drives (SSDs) or NVMe storage, provide the high read/write speeds needed to process large datasets efficiently.

The networking infrastructure is another critical component, with high-speed networks (e.g., 10 GbE or higher) ensuring efficient data transfer between storage and computation nodes. This is crucial for handling large datasets without bottlenecks. Securing networking is also essential for protecting sensitive biological data and complying with data privacy regulations.

Cloud computing platforms such as AWS, Google Cloud, and Azure offer scalable computing resources that can be adjusted on the basis of workload demands, providing flexibility and cost efficiency. These platforms also offer robust storage solutions and data management tools that facilitate data sharing and collaboration among researchers.

A compatible and optimized software environment is crucial for bioinformatics software, including tools such as R and Python, and specialized bioinformatics packages such as Bioconductor and SciPy. Efficient data management systems, including the SQL and NoSQL databases, are essential for storing, retrieving, and managing complex datasets.

Finally, the hardware requirements for multidimensional omics data analysis include high-performance central processing units (CPUs) and GPUs, large memory and storage capacities, high-speed networking, and a robust, scalable software environment. These resources ensure the efficient and effective processing of large and complex datasets, enabling comprehensive analysis and insights in omics research.

## Applications of Multi-Omics

### Multiomics-assisted decoding of genetic networks

Research advances in multiomics analysis of genetic networks have importantly contributed to our understanding of complex biological processes. For example, in cancer research, by integrating genomics, transcriptomics, epigenomics, and proteomics data, researchers can determine the molecular mechanisms of tumors [147]. The TCGA project has successfully identified key gene mutations and regulatory networks in various types of cancer through the integration of multiomics data [148]. These findings not only improve our understanding of the mechanisms underlying cancer development and progression but also provide potential targets for personalized therapies.

Another example is the GTEx project [110], which analyzes gene expression and genetic variation across different tissues and organs to uncover the functions and regulatory networks of genes in various biological systems. By integrating multiomics data, researchers have identified numerous disease-associated genes and their tissue-specific expression patterns. These discoveries are crucial for understanding the pathological mechanisms of complex diseases and for developing new therapeutic approaches.

The use of multiomics data to analyze genetic networks has made important progress in plant research. By integrating transcriptomic, metabolomic, and epigenomic data, researchers have identified key gene networks involved in plant growth, development, and stress resistance. For example, in Arabidopsis studies, the integration of multiomics data has led to the identification of key regulatory genes and metabolic pathways related to drought [149] and heat resistance [150], providing new insights and methods for breeding drought-tolerant crop species.

### Multiomics-assisted crop breeding

Traditional breeding methods are often inefficient, slow, and labor intensive. However, the application of multiomics in plant and animal breeding has shown promising results. Notable examples include advances in maize genetics and breeding, which have the potential to importantly accelerate the breeding process.

First, establishing multiomics databases facilitates gene discovery and data visualization analysis. For example, the ZEAMAP database for maize [151] includes data on the maize genome, population genomics, transcriptomes, open chromatin regions, chromatin interactions, high-quality genetic variants, phenotypes, metabolomics, and genetic maps. Similarly, MaizeNetome [152] integrates data from the genome, transcriptome, translatome, interactome, and other integrative omics sources.

Second, multiomics big data combined with machine learning can be used to predict maize yield. For example, a study [153] utilized multiomics data from 156 maize recombinant inbred lines, including 2496 SNPs, 46 imaging traits (i-traits) from 16 growth stages obtained through an automated phenotyping platform, and 133 major metabolites. Benchmark testing of various predictive models revealed that some machine learning methods, such as PLS, RF, and Gaussian process with radial basis function kernel (GaussprRadial), performed better in predicting maize yield, although the preferences for different methods varied slightly because of differences in i-trait, genomic, and metabolic data. Improved yield prediction likely stems from the ability of different methods to sort and filter data features, which are biologically relevant to processes such as photosynthesis or grain development. Ultimately, integrating multiomics data with RF machine learning methods further enhanced the accuracy of yield prediction, increasing it from 0.32 to 0.43.

Furthermore, by identifying key genes and regulatory elements, we can better understand the fundamentals of maize yield and use that understanding to provide new molecular markers and breeding strategies. For example, Hirsch et al. [154] utilized genomics and epigenomics techniques to study maize phenotypic traits. By integrating genome-wide association study (GWAS) and methylation data, they identified important genes and regulatory elements affecting maize yield and growth development. These findings offer new molecular markers and breeding strategies for maize improvement (Hirsch CN, et al. 2014. Insights into the maize pan-genome and pan-transcriptome. Plant Cell). Additionally, through transcriptomic, proteomic, and metabolomic analyses at different stages of grain development, researchers have identified several genes involved in phenylpropanoid biosynthesis that may be related to the large grain phenotype [155]. Finally, gene editing of favored genes/loci, as well as plant synthetic research [156,157], could enable fast and accurate crop breeding.

## Summary

**Fig. 3.**
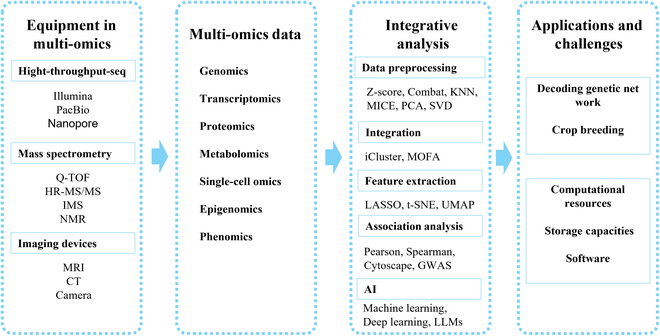
The workflow of data generation, integration, and application in multiomics analysis.

The study of genetic networks through multiomics analysis not only advances biomedical research but also has promising applications in agriculture and environmental science. By integrating various layers of omics data, researchers can gain a more comprehensive understanding of the complexity of biological systems, discover new biomarkers and therapeutic targets, and drive progress in precision medicine and crop improvement (Fig. 3). The integration of large-scale machine learning models in multiomics analysis represents an important improvement in our ability to understand complex biological systems. By leveraging advanced architectures such as transformers and GNNs and integrating diverse omics data, researchers can uncover new insights into disease mechanisms, identify novel biomarkers, develop personalized treatment strategies, or breed novel crops. This interdisciplinary approach, which combines computational expertise with biological knowledge, is paving the way for a new era of precision medicine and systems biology.
